# Cytochrome C and Caspase-3/7 are Involved in Mycophenolic Acid- Induced Apoptosis in Genetically Engineered PC12 Neuronal Cells Expressing the *p53* gene 

**Published:** 2014

**Authors:** Hassan Malekinejad, Masumeh Moradi, Johanna Fink-Gremmels

**Affiliations:** a *Department of Pharmacology and Toxicology, Faculty of Veterinary Medicine, Urmia University, Urmia, Iran. *; b *Department of Veterinary, Pharmacology, Pharmacy and Toxicology, Faculty of Veterinary Medicine, Utrecht University, Yalelaan 104, 3508 TD Utrecht, The Netherlands . *

**Keywords:** Apoptotic effect, Caspase 3/7, Cytochrome C, Mycophenolic acid (MPA), PC12 Tet Off cells (PTO)

## Abstract

Mycophenolic acid (MPA) is the active metabolite of mycophenolate mofetil. This study designed to investigate the mechanism of cytotoxicity of MPA on the genetically engineered PC12 Tet Off (PTO) neuronal cells with *p53 *gene. Alamar Blue (AB) reduction showed concentration-dependent cytotoxicity of MPA on PTO cells with IC_50_ value of 32.32 ± 4.61 μM. The reactive oxygen species (ROS) generation following exposing the cells to MPA showed a significant (p < 0.05) increase in the ROS production and in a concentration-dependent fashion. Involvement of Caspase 3/7 proteases and Cytochrome C release in the induction of DNA fragmentation are all hallmarks of MPA-induced apoptosis in PTO cells. Our data suggest that MPA exerts an apoptotic effect on PTO cells. Moreover, the apoptotic effect of MPA attribute to the elevation of ROS generation by which might trigger the cytochrome C release and the activation of Caspase 3/7 that ultimately results in DNA fragmentation.

## Introduction

Mycophenolic acid (MPA) is the active metabolite of mycophenalate mofetil (MMF), which is used as an immunosuppressant agent in organ transplantation (1). MPA also as a secondary metabolite is produced by *Penicillium spp. *(2). There are reports indicating the occurrence of MPA and MPA-producing fungi in human foods and animal feeds (3). 

The antiproliferative effect of MPA on lymphocytes and non-lymphatic cells such as mesangial cells has been reported. MPA by inhibition of inosine monophosphate dehydrogenase (IMPDH) leads to inhibiting *de novo *guanosine synthesis and by this mechanism suppresses the lymphocyte and mesangial cell proliferation (4). Another study suggests that the growth-inhibitory and pro-apoptotic effects of MPA are attributable to an inhibition of ribosomal RNA synthesis and nuclear disorganization in malignant cells (5). On the other hand, long term administration of MMF may result in unwanted effects of anemia due to inhibition of IMPDH activity in erythroid cells, gastrointestinal disorders, alteration of the plasma bioelements concentration, and allergic reactions (6, 7). 

There are increasing numbers of reports, which indicate an important role of *P53 *gene in mediating of apoptosis. The *p53 *gene is expressed at very low levels in normal cells. It is up regulated in response to DNA damaging agents such as UV- or γ–irradiation and by genotoxic compounds (8). Apoptosis induction via *p53 *pathway in T and B cells by using general apoptotic assays such as TUNEL and expression of Annexin-V has been demonstrated(9). As an experimental model, the PC12 cell line has been stably transformed with *p53 *Tet-Off gene and therefore expresses the tetracycline-controlled transactivator (tTA), in a stable way. 

There are reports indicating that long time administration of MMF in transplanted patients resulted in neuronal disorders such as progressive multifocal leukoencephalopathy (10). 

Previous studies also demonstrated that MPA induces apoptosis in various cells including islet cells of pancreas via mitogen-activated protein kinase activation and T lymphocytic cells via activation of caspase 3 (11, 12). 

There is, however, insufficient data about the cytotoxicity and mechanism of action of MPA on neuronal cells. It is also unclear in case of having apoptotic effects on neuronal cells, by which mechanism it is mediated. Hence, due to the long-term administration of MMF in organ transplanted patients and also in consideration of the natural occurrence of MPA on spoiled food and feed materials, we aimed to investigate the cytotoxic and apoptotic effects of mycophenolic acid on PC12 Tet Off cells carrying the *p53 *gene. Moreover, the possible apoptosis cascade of the MPA-induced toxicity was subjected to this investigation. 

## Experimental

*Chemicals*

2’, 7’ –Dichlorodihydrofluoroscein diacetate (H2DCF-DA) was obtained from Molecular probes (Leiden, The Netherlands). Tetracycline, mycophenolic acid (MPA), hygromycin B, phenyl methyl sulfonyl fluoride (PMSF), and monoclonal rat antibody for cytochrome C were purchased from Sigma Chemical Co. ST Louis, MO, USA. 

RPMI 1640 was supplied from Biocambrex, Belgium. Genecitin (G418), penicillin and streptomycin, Non Essential Amino Acids (NEAA), Foetal Calf Serum (FCS), and Trypsin EDTA were supplied by Invitrogen (Breda. The Netherlands). Tet system Approved Foetal Bovine Serum (Tet-Off FBS) and horse serum, were supplied from BD Bioscience Clontech, Palo Alto, CA, USA. Collagen Vitrogen-100 was obtained from Cohesion Technologies, INC. Palo Alto, California, USA, Caspase-3/7 assay kits were obtained from Promega (The Netherlands) and the DNA Laddering kit was purchased from Roche Diagnostics GmbH (Germany). Alamar Blue (AB) was obtained from Biosource International, Biosource, The Netherlands. Sucrose was purchased from BDH chemicals Ltd., Poole England. All other reagents were purchased from well-known chemical companies.

PC12-Tet Off (PTO) cells were a kind gift from Dr. Silvia Stingele, ECVAM, Italy.

*Cell culture*

PTO cells were grown in RPMI 1640 that supplemented with 10% horse serum, 5% Tet- Off FBS, 1 % L-glutamine, 150 μg/mL G418, 150 μg/mL hygromycin B, 2 μg/mL Tetracycline and 1% penicillin (100 units/mL), Streptomycin (100 μg/mL). Cells were incubated at 37 °C in a humidified atmosphere of 5% CO2 in air. Collagen Vitrogen-100-coated flasks and dishes were used during maintenance and experiments, respectively.

The collagen solution (1% -v/v- collagen Vitrogen-100; 1% -v/v- BSA, 10% HBS, 122 mM NaCL; 2.67 mM KCL; 9.4 mM Glucose; 14 mM NaH2PO4; 20 mM Hepes; pH 7.5) was added at least 3 hours at 37 °C prior to use. The coated flask was used immediately upon removal of the excess collagen. The *P53 *expression in PTO cells at protein level was tested in the presence and absent of 2 μg/mL tetracycline as a prerequisite of the study and the results showed that *P53 *is expressed in the absence of tetracycline 2-fold more than in the presence of tetracycline.

*Exposure of cells to MPA*

For AB reduction, caspase-3/7 activities and ROS generation assays PTO cells were seeded in 96-well tissue culture plates at density of 2 x 10^4^ cells/well. For Cytochrome C and DNA-laddering experiments, PTO cells were seeded in tissue culture dishes (60/15 mm), at density of 2-3 x 10^6^ cells /dish and in 4 mL medium with all supplements except tetracycline. Cells were incubated for 48 hours prior to adding the MPA for more expression of *p53 *gene. 

For all assays the old medium was removed and replaced with fresh medium containing the MPA at various concentrations (0, 10, 25, 50, 75 and 100 μM). The cells were incubated for an additional 24 hours, after which time caspase-3/7 activities, ROS production, Cytochrome C releasing and DNA-laddering assays were conducted. 

*Alamar Blue Reduction Assay*

Mitochondrial activities were measured following treatment with MPA serum by using AB reduction assay according to previous reports (13). In short; the commercial stock solution of AB was diluted 10-fold in RPMI-1640. Following incubation of the cells with the test compound, the medium was removed and the cells washed with warmed PBS. Medium containing AB (Diluted, 1:10) and cells were incubated for 3 hours at 37 °C. 

Thereafter, the fluorescence of the reduced AB was measured by using an excitation wavelength of 560 nm and an emission wavelength of 590 nm (Cytoflour 2300 Fluorescence Measurement System, Millipore Corp. Bedford, MA, USA). Cell viability was expressed relative to the controls (A- treated cells/ A-control cells) x 100. 

*Determination of ROS production*

Production of ROS was measured by using 2’, 7’, -dichlorodihydrofluorescein diacetate (H2DCF-DA), according to previously described method (14). The principle of this assay is that DCFH-DA diffuses through the cell membrane and is enzymatically hydrolysed by intracellular esterases to nonfluorescent dichlorofluorescein (DCFH). In the presence of ROS, this compound is rapidly oxidized to highly fluorescent dichlorofluorescein (DCF). Briefly, PTO cells were plated in 96-well culture plates. After 48 hours incubation, cells were washed with pre-warmed PBS and pre-incubated for 60-80 minutes with 20 μM H2DCF-DA in 50 μL krebs Ringer Phosphate Glucose solution (KRBG; 10 mM glucose, 10 mM Hepes, 140 mM NaCl, 5 mM KCl, 1.8 mM CaCl2, 1 mM MgSO4, pH 7.4). After mentioned incubation time H2DCF-DA solution was removed and cells rinsed with PBS and treated with different concentrations of MPA. Twenty four hours after treating of the cells with MPA, ROS production was measured using a spectofluorometer microplate reader (Fluostar optima, BMG Labtechnologies GmbH, Germany) at an emission wavelength of 538 nm and an excitation wavelength of 485 nm. Relative ROS production was expressed as an increase in fluorescence compared to fluorescence of the appropriate control (100%). H2O2 at 10 μM concentration was used as a positive control.

*Cytochrome C assay*

After the given treatment period, PTO cells were harvested with buffer containing 0.25 M sucrose, 0.1 mM EDTA, and 1 mM PMSF, and kept in ice for 15 minutes. Cells were disrupted using a glass homogeniser according to methods described previously (15). Following centrifugation at 10,000 x *g *for 15 min, 5 μg of cytosolic protein was fractionated by SDS-PAGE and analyzed by western blot analysis using a monoclonal rat antibody to Cytochrome C as described by Laemmli (1970) (16).Detection was performed by separately rinsing of monoclonal rat Anti-Cytochrome C as test protein (Bioscience) and rabbit monoclonal anti-β actin as a reference protein (Millipore) against mouse (IgG2bκ) for immunological staining. Protein concentrations of the PTO cells were determined based on Lowry *et al*. (1951) (17). Densitometric analyses of Western blots were performed using Molecular analyst software (version 1.5) from BioRad (Hercules. CA, USA) and the density of Cytochrome C bands were normalized against corresponding β actin bands. 

*Caspase-3/7 activities assessment*

Following exposure of PTO cells to MPA at various concentrations for 24 hours, 100 μL of homogeneous caspase-3/7 reagent was added on cells and cultured plates covered with aluminium foil were then gently shaken using a plate shaker at 300-500 rpm for 30 seconds to 1 min. Thereafter, cells were incubated for 8 hours. The fluorescence of treated cells was measured at an excitation wavelength of 498 nm and emission wavelength of 530 nm. Caspase-3/7 activities were expressed relative to controls as (A- treated cells/ A-control cells) x 100. 

*DNA-Laddering*

DNA of the MPA-treated PTO cells was purified based on apoptotic DNA Laddering kit manufacturer’s instructions (Roche Diagnostics, GmbH). Briefly, PBS and l lyses buffer (6 M guanidine-HCL, 10 mM Urea, 10 mM Tris-HCL, 20% Triton X-100 [v/v], pH 4.4) were added to cells and incubated for 10 min at 15-25 °C, after which time isopropanol was added to the samples. The filter and collection tubes (provided in the kit) were combined and samples pipetted into the upper reservoir. After centrifugation for 1 minute at 2860 x g (eppendorf centrifuge 5417 R) the eluate was discarded and the used collection tubes were combined with filters. 500 μL washing buffer (20 mM NaCl; 2 mM Tris-HCl; 80 % [v/v] ethanol: pH 7.5) centrifuged at 2860 x g for one minute, and after discarding the collection tube this washing phase was repeated and the residual wash buffer was removed by centrifuging for 10 seconds at 7550 x g, and was added to the upper reservoir. The filter tube was then inserted into a clean Eppendorf tube and for the elution of DNA, 200 μL of 72 °C pre-warmed elution buffer (10 mM Tris, ph 8.5) was added to the filter tube and centrifuged for one minute at 2860 x g. The eluted DNA was used directly or stored at –20 °C for subsequent analysis. 

DNA was quantified and a volume of the eluate corresponding to 2-3 μg DNA (15-17 μL of eluted DNA) was added to loading buffer (50% glycerol; 2 mM EDTA; 0.4 % bromphenol blue), and the DNA solution was run on a 0.8 % agarose gel for 60 minutes at 60 V constant voltage. λPST1 also was loaded as a marker for identification of amount of DNA. Gels were stained with ethidium bromide and visualized by UV light. 

*Statistic analysis*

Significance of differences between control and treatment with various concentrations of MPA in AB reduction, caspase-3/7 activities, Cytochrome C and ROS production assays determined using a one-way ANOVA and then Bonferroni test was used as posthoc test using Graph pad Prism (Version 4.1). Differences were considered significant if p < 0.05.

## Results

**Figure 1:**
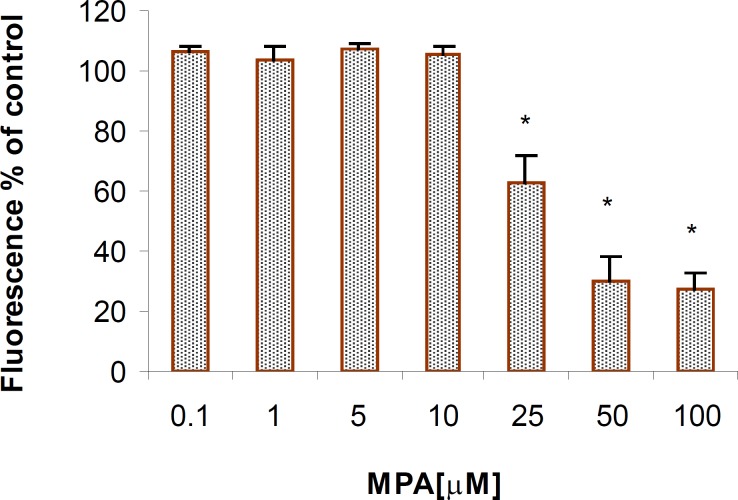
The cytotoxic effect of MPA on PTO cells (AB reduction); Significant differences are observed between the control and the cells that were exposed to concentrations higher than 25 μM of MPA. The columns indicate the mean of percentages of three independent experiments that were conducted in triplicate and error bars represent SEM

The mitochondrial activity, which has been measured by AB reduction, following exposure of the PTO cells to various concentrations of the MPA, declined and this reduction was in concentration-dependent fashion (Figure 1). The significant reduction in mitochondrial activity, which served as a marker for cell viability, was observed at concentrations higher than 25 μM MPA (p < 0.05).

**Figure 2:**
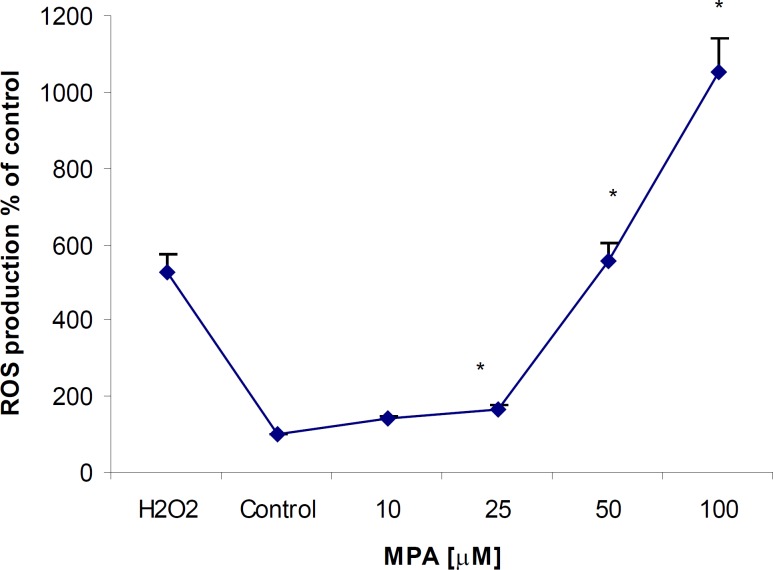
The effect of MPA on ROS generation in PTO cells; the cells were treated with different concentrations of MPA for 24 h. There are significant differences (p < 0.05) between the control and all tested concentrations higher than 25 μM of MPA. The data points indicate the mean of percentages of three independent experiments that were conducted in triplicate and error bars represent SEM

MPA increased ROS production after 24 h exposure period in PTO cells at all given concentrations; however it was found statistically significant (p < 0.05) at concentrations higher than 25 μM. ROS elevation showed a clear dose-dependency and maximum ROS production occurred at the highest given (100 μM) concentration (Figure 2). Comparing the level of produced ROS by MPA and H2O2 at the same concentration indicates that MPA is not strong as H2O2. The ROS generation in H2O2-exposed PTO cells was found 4 fold higher than that of MPA-treated cells.

**Figure 3:**
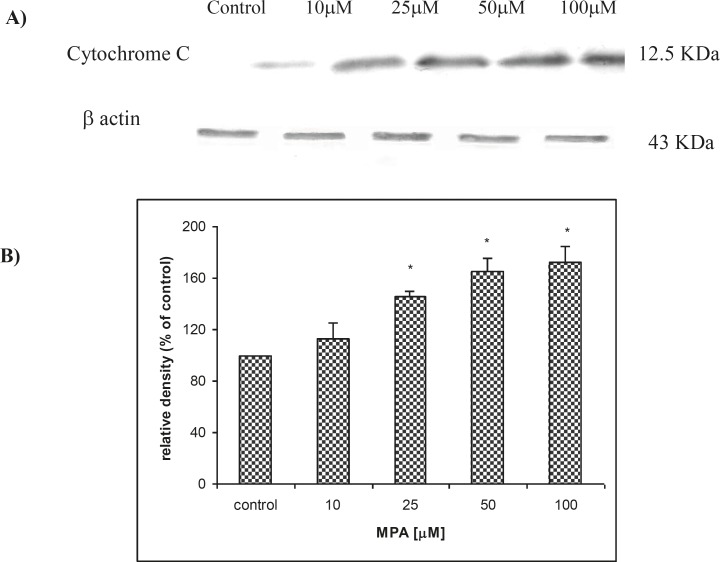
The effect of MPA on Cytochrome C release from treated PTO cells with MPA; **(A) **indicates the result of western blot analysis and shows that release of Cytochrome C is increased with increasing of MPA concentration and **(B) **shows the densitometric analysis of the Cytochrome C bands against the corresponding β actin bands

The releasing of Cytochrome C in PTO cells was evaluated by western blot analysis. As shown in Figure 3-A., releasing of Cytochrome C (12.5 kDa) followed a concentration-dependent fashion. The release of Cytochrome C was found significantly (p < 0.05) elevated at concentrations higher than 25 μM of MPA. The expression of cytochrome C in non-treated PTO cells in comparison to β actin was found remarkably lower. Densitometric analysis (Figure 3-B) revealed an increase in Cytochrome C release following treatment of PTO cells for 24 h with MPA, when normalized against corresponding β actin bands (43 KDa).

**Figure 4:**
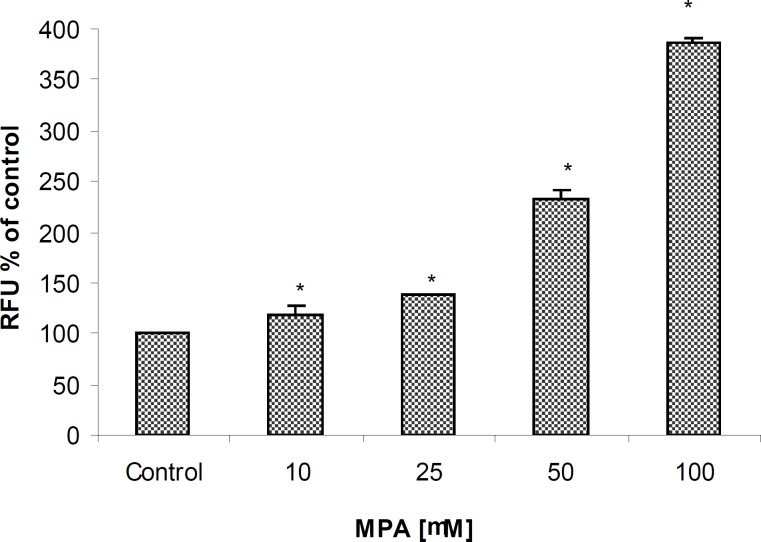
The effect of MPA on Caspase-3/7 activities in PTO cells; the cells exposed against different concentrations of MPA for 24 h. Statistically significant differences (p < 0.05) are seen between control and all studied concentrations of MPA.

Following 24 h treatment with MPA, an increase in caspase-3/7 activities as two executive members of caspases in PTO cells in a concentration-dependent manner was observed reaching at the highest examined concentration approximately 400% when compared to controls (Figure 4). 

Statistically significant differences (P < 0.05) between control and all examined concentrations (10, 25, 50, 75, and 100 μM) of MPA were already observed after 3 hours of incubation with homogeneous caspase-3/7 reagent. Unlike to previous markers, the elevation of caspase 3/7 activities at the lowest given concentration (10 μM) may represents the high sensitivity of assay. Additionally, a significant difference between two concentrations of 50 and 100 μM only was obtained in this assay which again indicating the highest sensitivity of measurement method. 

**Figure 5:**
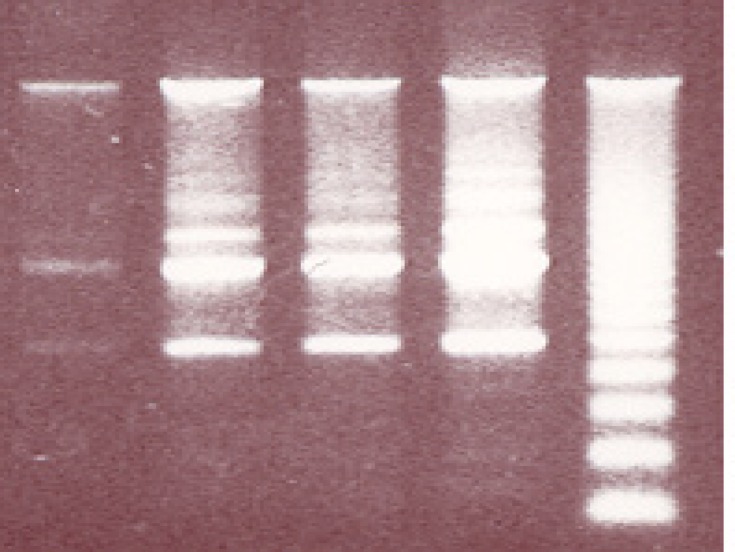
The effect of MPA on DNA fragmentation in PTO cells; In comparison with control remarkable differences are observed between the given concentrations of MPA following 24 h exposure to MPA. R indicates reference sample.

To evaluate whether the toxic effect of MPA on PTO cells results in complete apoptosis, the DNA fragmentation assay was conducted, as this is considered to be a typical index for apoptosis. MPA exerted DNA-laddering (fragmentation) in a concentration-dependent manner (Figure 5). In consideration of the results of the Cytochrome C release assay and also ROS production as an initiator factor for DNA damage, in the DNA-laddering experiments only the effective concentrations were chosen (25, 50 and 100 μM). 

## Discussion

This study reports that MPA exerts cytotoxic effects and induces apoptosis in PTO cells. The mechanism underlying the cytotoxicity and apoptosis in these cells is assumed to be initiated through the mitochondrial disturbances and ROS production, which followed by the elevation of Cytochrome C release from mitochondria as demonstrated. Moreover, it has been also explored in current study that Cytochrome C elevation may result in caspase 3/7 activation that in turn results in fragmentation of cellular DNA. 

The selection of PTO cells for this study was based on previous data indicating capability of *p53 *gene in apoptosis induction. There are data indicating that *p53 *along with other genes such as *JNK *and *p38 *(MAPK) are involved in apoptosis induction in neuronal cells (18). The cytotoxicity of MPA either from primary source of consumption of Penicillium-contaminated foods or from administration of MMF has been shown in different types of cells before (19). However, its cytotoxicity in neuronal cells and in particular in cells with *p53 *gene has not been evidenced. This report for the first time showed that MPA at concentrations up to 10 μM can produce a significant cytotoxicity in neuronal cells containing the *p53 *gene. Although it has generally been accepted that the mechanism of action of MPA on lymphocytes is cytostatic, its apoptotic effect on human T lymphocytes also has been reported. MPA widely known as a potent antiproliferative agent against lymphocytes, however its inhibitory effects on the glycosilation, expression and adhesion of functional molecules is also documented (20). As demonstrated by using AB-reduction test which normally indicates the cell viability based on mitochondrial function in producing the fluorescence metabolites, MPA declined remarkably cell viability at concentration of 25 μM, suggesting that mitochondrial dysfunction might be one of the initiating effects in the cytotoxity of MPA. Our results, however can not exclude other cytotoxic mechanisms of MPA in PTO cells as reported in T lymphocytes.

The second part of the present study was devoted to clarify the MPA-induced apoptotic response in PTO cells. Increased levels of ROS generation in MPA-exposed cells could be the potent signal that initiates apoptosis processes, as it has been reported by several studies on rodents (21, 22).

Another factor which mediates the MPA-induced apoptosis in PTO cells through mitochondrial pathway is the elevation of Cytochrome C release, as demonstrated in the current experiments. Assuming that MPA induces the apoptosis through mitochondrial pathway, it should be taken in account that changes in the permeability of the mitochondrial outer membrane cause an outflow of Cytochrome C, probably mediated by Bax (a channel-forming protein located at the outer membrane of mitochondria) and inhibited by members of the *Bcl-2 *protein family (23, 24). We showed that after 24 h exposure of PTO cells to MPA, a remarkable increase of Caspase 3/7 activities occurs. It has been documented that Cytochrome C interacts with Apaf-1 protein, dATP, and multiple molecules of pro-caspase-9 to generate an active complex of the apoptosome that activates caspase-9. Activation of caspase-9 initiates the so-called caspase cascade that induces apoptosis. In the mentioned cascade, crucial points are the activation of caspase-3 and caspase-7. The role of caspases-3 and -7 in Apaf-1 proteolytic cleavages in cisplatin-induced apoptosis in melanoma cells and in macrophages following infection with *E. coli *have been reported before (25). The obtained results suggest that MPA-induced apoptosis is a caspase-dependent processes 

The last phase of apoptosis is the post-mortem phase of the cell, in which the cell’s chromatin condenses and its DNA is degraded. MPA-induced apoptosis was confirmed by the DNA-laddering assay. Disintegration of nuclear DNA into oligonucleosomal fragments represents a classical manifestation of apoptosis (26). The discovery of caspase-activated DNase (CAD) and its inhibitor ICAD has provide a direct link between caspase and apoptotic DNA disintegration. Treatment with caspase-3 led to the cleavage of DNA fragmentation factor subunit of 45 kDa (DFF- 45) and induction of apoptosis (27). Both of the ICAD caspase recognition sites (amino acid positions 117 and 224), must be cleaved by caspase-3 and –7 (but not with caspase 1, 2, 4-6, and 8) to activate CAD. As soon as apoptosis is initiated, activated caspase-3 cleaves ICAD-L (full-length form of ICAD), and releases CAD, which is capable of degrading the chromosomal DNA (28). 

*In conclusion*, our data suggest that the cytotoxic mechanism of MPA in PTO cells is related to its apoptotic properties. The MPA-induced apoptosis is apparently initiated by mitochondrial dysfunctioning, followed by increased Cytochrome C release and caspase 3/7 activation and completed by DNA fragmentation. Therefore, as the MPA is the main and active metabolite of long-term administered immunosuppressive agent MMF for the preventing of transplanted tissues rejection, thus our results raise concern about the possible neuropathies due to MMF administration. In addition, as MPA is a food and feed contaminant, monitoring of MPA in these commodities should be initiated and results subjected to risk assessment. 
